# Decoding the brain: Unveiling comprehensive cellular atlases through multiomics mega-data

**DOI:** 10.1016/j.xinn.2024.100637

**Published:** 2024-05-06

**Authors:** Qianwen Wang, Wenli Tang, Lukui Chen, Lin Deng, Guangchuang Yu

**Affiliations:** 1 Department of Bioinformatics, School of Basic Medical Sciences, Southern Medical University, Guangzhou 510515, China; 2 Department of Neurosurgery, Southern Medical University Hosptial of Integrated Traditional Chinese and Western Medicine, Southern Medical University, Guangzhou 510515, China

## Main text

**Figure 1:**
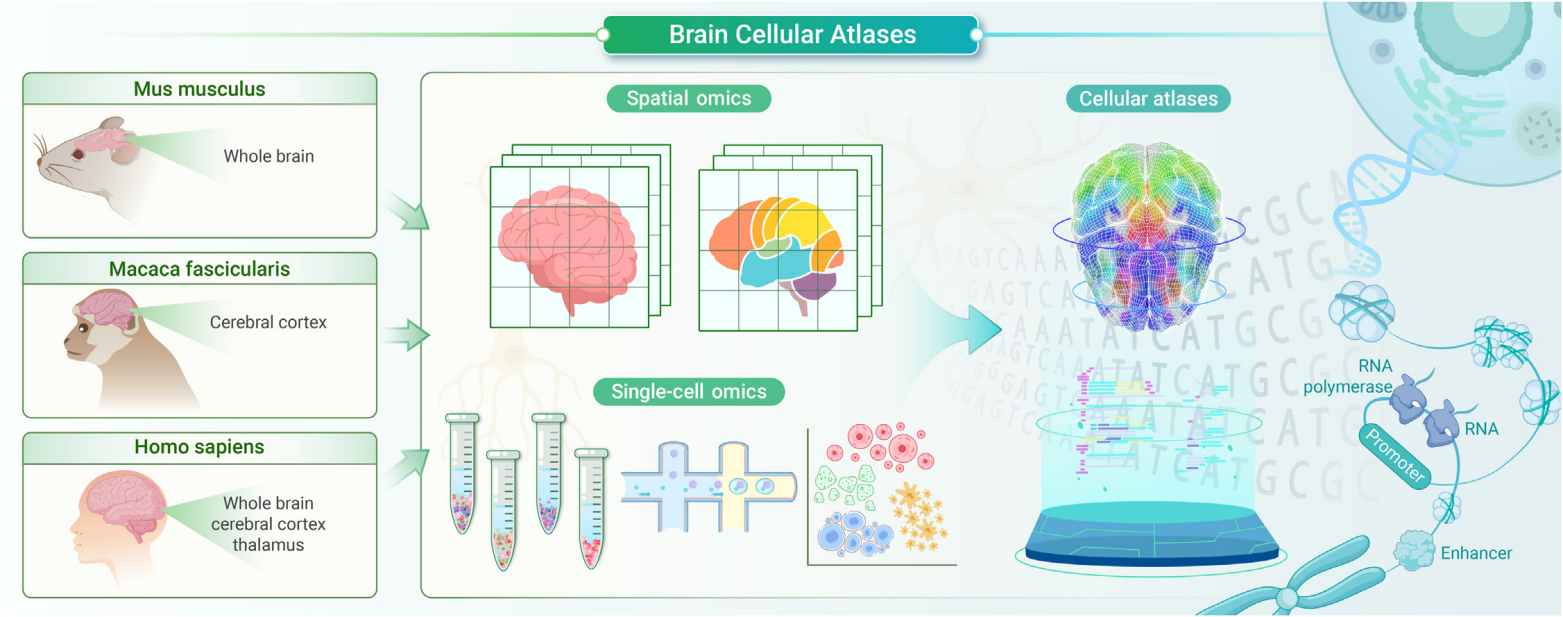
Cellular atlas of mouse, macaque, and human brains unveiled through large-scale single-cell and spatial omics

The pursuit to comprehend the intricate nature of the brain, an organ of unparalleled complexity, remains a persistent endeavor in the field of neuroscience.^1^ Recent strides by the National Institutes of Health’s BRAIN Initiative – Cell Census Network (BICCN) have yielded comprehensive cellular atlases for mice and humans,^2^^,^^3^ published in *Nature* and *Science*, respectively. Additionally, a 3D cell-type atlas of the macaque brain, contributed by *Cell*, offers profound insights into non-human primate cortical intricacies.^4^ These collaborative initiatives, driven by the US’s BICCN and China’s Brain Project, represent a significant leap forward in unraveling the cellular intricacies of the brain, culminating in groundbreaking exploration across mice, non-human primates, and humans. The confluence of these works serves to elucidate the intricate cellular landscapes of the brain through a multiomics approach, signifying a pivotal advancement in neuroscience research toward comprehensive understanding and collaborative exploration (Figure 1).

## Unveiling the molecular landscape of the whole mouse brain: Insights from cellular atlases

Within the expansive realm of mammalian brains, where millions to billions of cells intricately form networks essential for myriad functions, the exploration of the mouse brain’s cellular terrain has reached unprecedented depths, courtesy of the revolutionary cellular atlases offered by the BICCN.^2^

The collaborative endeavors of the BRAIN Initiative have been pivotal in deciphering the intricate spatial organization of molecularly defined cell types within the mouse brain.^2^ Representing a quantum leap in neuroscience, these atlases, facilitated by innovative technologies of single-cell RNA with 7 million cells and spatial sequencing with 10 million cells, have unveiled a complex landscape comprising a whole mouse brain. This comprehensive understanding reveals unexpected nuances in cell-type diversity across different brain regions. Certain regions surprisingly exhibit a high diversity of cell types, while others manifest fewer types with greater divergence. Advancing with single-cell sequencing of epigenomics, the classification power of transcription factor genes emerges as a key player in defining the brain’s cell-type diversity, through integrative analysis of 2.3 million cells indicating that specific combinations of expressed transcription factors play a pivotal role.^2^ Moreover, the coordinated relationship between the molecular identity of neurons and their epigenomics projection specificity offers valuable insights into the epigenomic landscape that shapes the interaction between genetic activity in different brain regions.^2^ This deeper understanding of the cellular architecture of the mouse brain not only enhances our knowledge of brain structure and function but also holds promise for exploring the molecular basis of neurodegenerative diseases and psychiatric disorders.

As researchers navigate through these uncharted territories of multiomics mega-data, the cellular atlases not only contribute to our understanding of the brain but also signify a paradigm shift in the approach to neuroscience. The collaborative metadata utilization serves as a guiding beacon, emphasizing the multidimensional exploration needed to unravel the mysteries of the brain in this new era of scientific discovery.

## Charting primate cortical complexity: Revelations from spatially resolved single-cell transcriptome atlases

The exploration of non-human primates serves as a pivotal link, bridging the understanding between mice and humans and providing invaluable insights into the evolutionary trajectory of the primate brain. A significant contribution to this endeavor comes from the publication in *Cell*, which unveils a groundbreaking spatially resolved single-cell transcriptome atlas of the macaque cortex, presenting a comprehensive cell-type taxonomy for the entire cortex.^4^

Leveraging large-scale single-nucleus RNA sequencing and spatial transcriptomic analysis, around 6 million cells meticulously mapped the distribution of 264 transcriptome-defined cortical cell types. Particularly noteworthy are the primate-specific cell types identified in layer 4 through cross-species analysis, underscoring the critical importance of accounting for evolutionary divergence in studies focused on the brain. The nuanced insights derived from the cortical layer and region preferences of different cell types provide a sophisticated understanding of the organizational principles and evolutionary dynamics shaping primate cortical regions. As we navigate through the layers of the macaque cortex, guided by the comprehensive cell-type taxonomy, we gain not only a deeper understanding of the intricacies of primate brain structure but also valuable insights into the shared and distinct features that define different species within the primate lineage. This atlas not only enriches our comprehension of the intricate cellular organization within the macaque cortex but also illuminates key aspects of primate brain evolution, development, aging, and pathogenesis. This knowledge is not merely an academic pursuit but has far-reaching implications for understanding the origins of human cognition, behavior, and neurological disorders that manifest across the primate spectrum.

In summary, the study of non-human primates, as illuminated by the spatially resolved single-cell transcriptome atlas in *Cell*, contributes significantly to the broader narrative of primate brain evolution. The collaborative and multidimensional approach adopted in this research sets a precedent for unraveling the complexities of the primate brain and opens avenues for future investigations into the fundamental principles governing brain diversity across species.

## Deciphering the intricacies of human brain: Revelations from comprehensive single-cell studies

The exploration of the human brain at the cellular level is integral to advancing our understanding of its complex architecture and functional dynamics. A series of collaborative studies, presented in the context of the BICCN provide a meticulous single-cell compendium of the human brain covering the whole brain, cerebral cortex, and thalamus.^3^

The groundbreaking study explored nearly 100 anatomical locations within the adult human brain, shedding light on the intricate distribution of over 3.3 million cells with distinct cell types across diverse brain regions.^3^ Employing a meticulous top-down methodology, researchers isolated postmortem tissue from various brain regions, facilitating iterative clustering of cells. This rigorous process resulted in a remarkable classification, revealing 31 superclusters, 461 clusters, and an astonishing 3,313 subclusters. While certain brain regions exhibit unique cell types, many regions primarily differ in the relative proportions of a common set of cell types. Additionally, another study examined eight areas of the adult human cerebral cortex over 1.2 million cells, demonstrating that while most areas contain similar cell classes, they mainly differ in the proportions of each type and the localization of non-neuronal cells within the cortical layer.^3^ This ambitious initiative challenges existing assumptions and emphasizes the need for comprehensive tissue sampling beyond the human brain.

By integrating single-cell and spatiotemporal omics, an in-depth transcriptomic investigation of over 160,000 cells from the developing human thalamus, a critical region for sensory information processing, provides essential insights into cellular diversification during critical developmental stages.^2^ This study elucidates the molecular identities and spatial arrangement of cell types within the developing human thalamus, shedding light on a less-explored GABAergic neuronal population that could potentially influence human evolution.

In sum, the collaborative endeavors of the BICCN, showcased in these studies, signify a notable stride forward in elucidating the cellular intricacies of the human brain. By unraveling the nuances of cell types, their proportions, and regional diversity, these findings not only contribute to the mechanistic understanding of human brain evolution but also pave the way for the development of experimental models tailored to study neurological diseases. This collective effort aims to establish a foundational understanding of how the human brain is constructed and functions, ushering in a new era of precision experimental investigations into the etiology of neurological disorders.

## Advancing neuroscience through collaborative multiomics mega-data

The collaborative multiomics mega-data, especially the integration of spatial and single-cell omics technologies, is reshaping our understanding of the brain’s complexities, with researchers synthesizing metadata to construct comprehensive cellular atlases across species.^2^^,^^3^^,^^4^ This approach not only unveils the intricate cellular terrain of the brain but also offers a multidimensional grasp of its complexity, revolutionizing our approach to neurological challenges and paving the way for personalized interventions.

Looking ahead, while significant progress has been made in generating the brain cellular resource, there are still untapped opportunities for further exploration within the realm of neuroscience applications. The increasing volume of data presents both challenges and opportunities for the field, as researchers strive to harness this wealth of information to refine diagnostic tools, treatment strategies, and personalized interventions for neurological disorders.^5^ By delving deeper into the spatial and single-cell aspects of brain research, researchers are uncovering transformative insights that have the potential to reshape our understanding of neurological disorders and enhance patient care.

In conclusion, the collaborative efforts in neuroscience research have laid a strong foundation for addressing specific neurological problems and advancing scientific understanding. By leveraging spatial and single-cell omics technologies alongside metadata-driven insights, researchers are poised to unlock new discoveries that can improve diagnostic capabilities, treatment strategies, and ultimately, patient outcomes. The utilization of metadata not only guides current exploration but also sets the stage for future directions in neuroscience, offering promising avenues for further advancements in brain research and patient care.
